# Studying Closed Hydrodynamic Models of “*In Vivo*” DNA Perfusion in Pig Liver for Gene Therapy Translation to Humans

**DOI:** 10.1371/journal.pone.0163898

**Published:** 2016-10-03

**Authors:** Luis Sendra, Antonio Miguel, Daniel Pérez-Enguix, María José Herrero, Eva Montalvá, María Adelaida García-Gimeno, Inmaculada Noguera, Ana Díaz, Judith Pérez, Pascual Sanz, Rafael López-Andújar, Luis Martí-Bonmatí, Salvador F. Aliño

**Affiliations:** 1 Departamento de Farmacología, Facultad de Medicina, Universidad de Valencia, Valencia, Spain; 2 Servicio de Radiología y Grupo de Investigación Biomédica en Imagen GIBI239, IIS La Fe y Hospital Universitario y Politécnico La Fe, Valencia, Spain; 3 Unidad de Farmacogenética, IIS La Fe y Área Clínica del Medicamento, Hospital Universitario y Politécnico La Fe, Valencia, Spain; 4 Unidad de Cirugía Hepatobiliopancreática y Trasplante, Hospital Universitario y Politécnico La Fe, Valencia, Spain; 5 CIBER e Instituto de Biomedicina de Valencia, CSIC, Valencia, Spain; 6 Servicio Central de Soporte a la Investigación Experimental (SCSIE), Universidad de Valencia, Valencia, Spain; 7 Servicio de Anatomía Patológica, Hospital Universitario y Politécnico La Fe, Valencia, Spain; 8 Unidad de Farmacología Clínica, Área Clínica del Medicamento, Hospital Universitario y Politécnico La Fe, Valencia, Spain; Justus Liebig Universitat Giessen, GERMANY

## Abstract

**Introduction:**

Expressing exogenous genes after naked DNA delivery into hepatocytes might achieve sustained and high expression of human proteins. Tail vein DNA injection is an efficient procedure for gene transfer in murine liver. Hydrodynamic procedures in large animals require organ targeting, and improve with liver vascular exclusion. In the present study, two closed liver hydrofection models employing the human alpha-1-antitrypsin (*hAAT*) gene are compared to reference standards in order to evaluate their potential clinical interest.

**Material and Methods:**

A solution of naked DNA bearing the *hAAT* gene was retrogradely injected in 7 pig livers using two different closed perfusion procedures: an endovascular catheterization-mediated procedure (n = 3) with infrahepatic inferior vena cava and portal vein blockage; and a surgery-mediated procedure (n = 4) with completely sealed liver. Gene transfer was performed through the suprahepatic inferior cava vein in the endovascular procedure and through the infrahepatic inferior vena cava in the surgical procedure. The efficiency of the procedures was evaluated 14 days after hydrofection by quantifying the hAAT protein copies per cell in tissue and in plasma. For comparison, samples from mice (n = 7) successfully hydrofected with hAAT and healthy human liver segments (n = 4) were evaluated.

**Results:**

Gene decoding occurs efficiently using both procedures, with liver vascular arrest improving its efficiency. The surgically closed procedure (sealed organ) reached higher tissue protein levels (4x10^5- copies/cell) than the endovascular procedure, though the levels were lower than in human liver (5x10^6- copies/cell) and hydrofected mouse liver (10^6- copies/cell). However, protein levels in plasma were lower (p<0.001) than the reference standards in all cases.

**Conclusion:**

Hydrofection of hAAT DNA to “in vivo” isolated pig liver mediates highly efficient gene delivery and protein expression in tissue. Both endovascular and surgically closed models mediate high tissue protein expression. Impairment of protein secretion to plasma is observed and might be species-related. This study reinforces the potential application of closed liver hydrofection for therapeutic purposes, provided protein secretion improves.

## Introduction

The possibility of expressing exogenous genes after naked DNA delivery was first described in late 1990s [1, 2]. Subsequently, hydrodynamic gene transfer was experimentally introduced [3, 4] as the rapid injection through the tail vein in mice of a large volume of saline solution containing the gene of interest. This intravascular hydrodynamic procedure for transferring genes is safer and harmless when compared with viral gene therapy [5].

Not just tissue protein but sustained and high expressions of human proteins in mouse serum have been reported. Promising results and successive advances were achieved, with therapeutic serum expression of the human alpha-1-antitrypsin gene (hAAT) for periods of over 6 months in mice [6]. Since transferring the hydrodynamic gene injection procedure to the clinical setting appeared promising, other research groups studied different approaches to adapt the procedure to larger animals by using lower volumes, since these would not tolerate the hemodynamic stress [7] derived from doubling the circulating volume in a matter of seconds, as occurred in mice [8, 9]. Given that transferring the procedure to larger animals could raise methodological, molecular and/or species-related problems, different alternatives were tested. To reduce the prohibitively large injection volumes, percutaneous radiologically targeted catheterization procedures were used in rats [10, 11], rabbits [12] and dogs [2]. In recent years, interest has focused on achieving the expression of human genes in pig liver through organ perfusion isolation by either interventional catheterization [13–20] or surgical hydrofection [21–23]. DNA hydrofection should be understood as a procedure characterized by the locoregional vascular administration of an aqueous DNA solution for therapeutic purposes. Hydrofection is an adaptation, with translational potential, of the successful hydrodynamic transfection procedure performed in mice, but avoiding the hemodynamic side effects that make it incompatible for application in large species (i.e., pigs and humans). Despite the efforts to adapt the procedure, none of the different approaches reported by different groups have yielded positive outcomes.

Standardization of interventional hydrofection delivery of genes to improve efficacy involves evaluating the influence of plasmid concentration, injected volume, flow rate, and flow direction [20] (anterograde through portal vein or retrograde through the suprahepatic inferior vena cava). Since hydrodynamic gene transfer employing enhanced fluorescence protein (eGFP) gene in watertight human liver segments [24] “*ex vivo*” mediated efficient protein expression, we considered it necessary to determine whether hydrodynamic gene delivery in isolated pig liver “*in vivo*” improves the efficiency of gene transfer. In the present study, liver vascularization was blocked “*in vivo*” using two procedures: an endovascular procedure employing three balloon catheters to simultaneously block the supra- and infrahepatic inferior vena cava and portal vein; and a surgical procedure involving ligation of the hepatic artery, inferior vena cava and portal vein. Liver vascular isolation allowed increasing the proportional flow of plasmid solution in a reduced area/target organ.

In this study we show that hydrofection, by means of surgical and endovascular procedures, has been efficiently performed, following three different safe and technically feasible methods for clinical use. The three methods also yielded protein expressed from the transgene in liver tissue. However, this protein was not able to reach the bloodstream. This leads us to hypothesize that the problem is related to the use of the swine model, and would be solved on translating the techniques to humans.

## Material and Methods

With regard to human samples, the study protocols complied with the ethical guidelines of the Declaration of Helsinki, and written informed consent was given. The study was approved by the Ethics Committee of the Hospital Clínico Universitario de València (Valencia, Spain). The Biological Research Ethics Committee of both the Instituto de Investigación Sanitaria La Fe and the Universitat de València (Valencia, Spain) approved the experiments. All animals were cared for according to the “Guide for the Care and Use of Laboratory Animals” (National Institutes of Health publication 86–23, revised 1985).

## Animals

Pigs were individually housed in pigsties. Seven female pigs (18–22 kg) were used. Three underwent a closed endovascular procedure while four underwent a surgical procedure. Pigs were not different regarding weight (20.6 kg vs. 19.3 kg average weight, respectively) or age (3 months). Anesthesia was induced with ketamine (Imalgene® 100, Merial France; 5–10 mg/kg, im), midazolam (Hospira® 1 mg/ml, Madrid, Spain; 0.3 mg/kg, im) and propofol (Lipuro® 2%, Braun, Melsungen, Germany; 4–6 mg/kg, iv), and was maintained with isoflurane (Isoflo®, Abbott laboratories, Madrid, Spain; 2.5% via the inhalatory route). Muscle relaxation was induced with vecuronium bromide (Norcuron® 10 mg; 0.08 mg/kg, iv). Morphine (0.4 mg/kg, iv) was administered for intraoperative analgesia, and buprenorphine (Buprex®, Schering-Plough, Madrid, Spain; 0.02 mg/kg, iv) was used for postoperative analgesia. Vital functions were monitored throughout the intervention. The pigs were sacrificed 14 days after the operation using potassium chloride (Braun 2 mEq, 20 mEq, iv), after sedation. Blood samples (2 ml) were collected from an ear vein at 0 h (before plasmid injection), and 1, 2, 4, 7, 10 and 14 days after injection, before sacrifice. The liver was extracted, and representative tissue samples of each lobule were collected for further analysis.

Male C57BL/6 mice (B&K Universal Ltd.) were used for *in vivo* experiments of hAAT gene transfer. The animals were maintained under standard laboratory conditions and housed 3–4 per cage. Anesthesia was induced and maintained with isoflurane (Isoflo®, Abbott laboratories, Madrid, Spain; 5% and 2%, respectively, via the inhalatory route). Three-ml latex-free syringes (Beckton Dickinson) and 25G syringe needles were employed to perform the hydrodynamic injection as previously described (2.0 ml saline solution containing 40 μg of plasmid at flow rate of 0.3 ml/s). Mice were sacrificed by cervical dislocation 7 days after gene transfer, and liver tissue and blood samples (200 ml) from tail vein were taken for further analyses.

## Catheterization

Different introducers and multipurpose catheters were used for jugular, femoral and portal venous access (Gore®); angioplasty balloons for vena cava (4/40) and portal vein occlusion (4/32) (Coda® Cook® Birmingham); and specially adapted 8Fr (French) balloon introducers for genic solution infusion.

**Fig 1:**
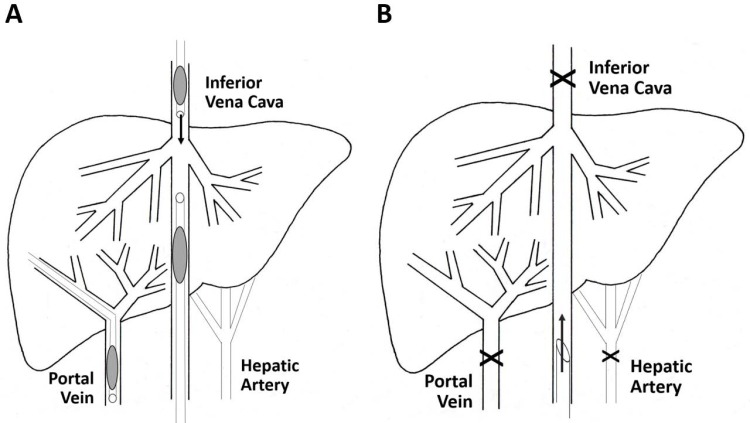
Open and closed endovascular catheterization procedure. The endovascular closed procedure is represented in Fig 1A. A schematic representation of the procedure is provided. An 8Fr balloon introducer and a 10Fr balloon catheter were placed in the vena cava, limiting entrance to the liver. Another transhepatic 10Fr balloon catheter was placed in the portal vein to block outflow. Fig 1B provides a schematic representation of the surgery closed model. After midline laparotomy, the liver is exposed and its vasculature (veins and artery), except the infrahepatic inferior vena cava, is referenced and ligated, thus completely sealing the organ. Retrograde gene transfer is performed through an 11 Fr catheter.

In the *closed endovascular procedure* (three pigs), the whole liver was retrogradely perfused after blocking both liver venous inflow and outflow during plasmid perfusion. Two (8Fr and 10Fr) catheters with balloon occlusion were placed at both suprahepatic and infrahepatic vena cava segments after jugular and femoral vein catheterization. A 10Fr balloon occlusion catheter was transhepatically placed in the main portal branch (Fig 1A). After solution perfusion, the liver was kept under total venous vascular exclusion for no more than 5 minutes.

The *closed surgical procedure* (four pigs) used three types of sutures: BiosynTM 5/0 (COVIDIEN) for vena cava, ProleneTM 6/0 (ETHICON) for portal vein, and DexonTM 2/0 (COVIDIEN) for closing the abdominal wall. Staples were used to close the skin.

A complete midline laparotomy was carried out, exposing all the abdominal organs. Liver vasculature was exposed and referenced. The clamping sequence was as follows: first the hepatic artery, then the portal vein and finally the infrahepatic vena cava, to fully interrupt hepatic inflow (Fig 1B). The suprahepatic vena cava was clamped last, to secure total hepatic vascular exclusion. A longitudinal incision was made on the anterior surface of the cava vein to insert the perfusion cannula. After solution perfusion, the liver was kept under total vascular exclusion for no more than 5 minutes. Progressive declamping was carried out in the reverse sequence, first allowing liver outflow and finally inflow through the portal and cava veins.

## *In vivo* gene transfer

In both closed models, a solution containing the hAAT plasmid (20 μg/ml) was retrogradely injected (200 ml at 20 ml/s) after partial (radiological) or complete (surgical) vasculature closure. Plasmid pTG7101 (18.6 kilobase, kb) contains a 16.5 kb genomic fragment of the hAAT gene, a 1.8 kb genome sequence upstream promoter, the promoter and full length of the human AAT gene, and a 3.2 kb downstream gene. For control purposes, liver tissue samples proceeding from an eGFP-transfected pig were employed. The eGFP gene transfer was performed by the surgical closed procedure under the conditions described above. The plasmid p3c-eGFP (6.45 kb), containing the enhanced green fluorescent protein complementary DNA (cDNA) driven by pCMV (cytomegalovirus) promoter, was constructed by cloning eGFP into the HindIII site of pcDNA3 (Invitrogen, Barcelona, Spain), excised from peGFP-N1 (4.7 kb) plasmid vector (Clontech Laboratories, Saint-Germain-en-Laye, France).

## Biological and pathological analysis

The quantification of hAAT DNA, RNA and protein was performed as previously described by our group [23], and data were expressed as copies of each molecular species per normalized cell. Liver tissue samples (< 1 mm thick) from all pigs were collected for molecular analysis and stabilized in RNAlater® solution (Sigma-Aldrich, Madrid, Spain). The samples were cut into small pieces, homogenized, and DNA, RNA and protein were extracted and purified. RNA was retrotranscribed to cDNA. The amount of DNA and cDNA was determined employing quantitative real-time polymerase chain reaction (qPCR) and power SYBR Green PCR master mix (Life Technologies). Oligonucleotides were designed with Primer Express software (Life Technologies). Data were plotted on a standard curve especially prepared with quantified human DNA. Curve sensitivity allows quantification of up to 5x10^-5 copies of hAAT DNA per cell and 2x10^-3 copies of hAAT RNA per cell. Data are expressed as copies/normalized cell considering the weight of the pig diploid genome [25] (5.4 pg) for gene delivery, and the average content of RNA per cell in a mammalian hepatocyte (20 pg) for gene transcription. Gene delivery and gene transcription indexes were determined and presented as copy number per normalized cells. Translation index indicates the total copy number of hAAT protein molecules per “normalized” cell. The approximate amount of protein per normalized cell (0.5 ng) was used for evaluating the protein translation rate per cell [26–28]. Whole protein content was determined in each sample with the NanoOrange protein quantitation kit (Invitrogen, CA, USA). Specific hAAT protein enzyme-linked immunosorbent assay (ELISA) and Western blot were performed [29, 30]. The assay was performed in 96-well microtitration plates (CoStar). In brief, goat anti-hAAT (0.2 mg/well) and goat anti-hAAT peroxidase conjugate (1.5 mg/well) from Sigma (Madrid, Spain) and ICN (Aurora, OH, USA) were used as capture and detecting antibodies, respectively. The enzymatic reaction was induced with o-phenylenediamine (0.4 ng/ml, in citrate-phosphate buffer, pH 5) with 30% H2O2 (1.5 ml/ml). The reaction was stopped 2.5 min later by adding 2 M H2SO4. The standard curve obtained was linear from 1.25 to 80 ng/ml, with correlation rate >0.99. Sample data were always within this range. The results, obtained as concentration, were converted to molecule number per cell considering the molecular weight of the hAAT protein (50 kDa), and quantifying the amount of total protein in each individual sample. The whole hAAT gene decoding process (DNA, RNA and protein) in hydrofected pigs was compared with two standards: hAAT transfected mice and normal human liver.

Quantitative data are expressed as copies of each species per normalized pig cell (DNA: 5.4 pg; RNA: 20 pg; protein: 0.5 ng), resulting in the following indexes:

- Gene delivery index: (no. hAAT DNA copies/pg of total DNA) * 5.4
- Gene transcription index: (no. hAAT mRNA copies/pg of total RNA) * 20
- Gene translation index: (no. hAAT protein molecules/pg of total protein) * 500

For immunohistochemistry, liver samples were incubated in antibody solution (1:300 diluted rabbit anti-hAAT, Sigma-Aldrich, Madrid, Spain) at 37°C for 1 h. Detection was carried out using the LSAB-2 peroxidase kit (DAKO). Tissue was counterstained with hematoxylin before dehydration.

Aspartate aminotransferase (AST) and alanine aminotransferase (ALT) levels were determined by automatized serum analysis [13].

## Standards

Seven male C57BL/6 mice (B&K Universal Ltd.) were used for *in vivo* hAAT gene transfer as reference standards for hAAT tissue and plasma levels. Hydrodynamic injection (40 μg of plasmid, flow rate 3 ml/s) and molecular determination of hAAT in liver tissue and blood samples (200 ml) from the tail vein were performed [6] 7 days after injection.

Four healthy human liver segment specimens, without hAAT deficiency, were obtained from surgical resections of metastatic cancer patients [24]. Tumor-free samples were taken for molecular analyses.

For molecular analysis, mice and human samples were processed in the same way as the treated pig samples, in order to compare molecular genetic expression.

## Statistical analysis

Data were summarized as the mean ± SD (standard deviation). Differences in protein copies/cell between groups were assessed using linear mixed models with individuals as random factor to account for the repeated measures design. Differences regarding serum levels of hAAT protein over the 14 days were assessed using a linear regression model with the area under the curve (AUC) as dependent variable. Differences in expression levels among species were assessed using a linear mixed model with expression level as response variable and species, sample type (DNA, RNA, protein in tissue or plasma) and their interaction as explicative variables. Ninety-five percent confidence intervals were computed for each estimation using bootstrapping. Statistical analyses and graphs were performed using R (version 3.1.2) with the lme4 R-package (version 1.1–7), and Prism 5 (GraphPad Software, San Diego, CA, USA), considering p<0.05 as statistically significant.

## Gold nanoparticle synthesis

All chemicals were purchased from Sigma-Aldrich (Madrid, Spain) and were used as received, without further purification. Gold nanoparticles were synthesized following a modification of the Turkevich method. Typically, 0.4 g of HAuCl4.3H2O was dissolved in 90 ml of water in a two-neck round flask (1.3 mM HAuCl4). The resulting solution was heated to boiling and refluxed. Then, 9 ml of a 47.2 mM sodium citrate solution (0.125 g sodium citrate in 9 ml water) was preheated and quickly added. The solution turned from yellow to black and to deep red. After the color changed, the solution was refluxed for 20 min. Then, the heater was turned off and the solution was stirred until it reached room temperature. The resulting solution was diluted with water to a final volume of 500 ml. Gold nanoparticles were characterized by transmission electron microscopy (TEM) (Jeol 1010, Tokyo, Japan). The average diameter and the size distribution of the gold nanoparticles were determined from TEM images by counting at least 300 nanoparticles using Image Pro Plus software from Media Cybernetics (Bethesda, MD, USA).

## Transmission electron microscopy (TEM)

Pig livers (n = 2) were injected (volume 200 ml) with a solution of citrate buffer containing 10^12 gold particles (4 nm and 15 nm in diameter) per ml following the surgically closed procedure to study the liver distribution of gold nanoparticles after injection. Then, small tissue pieces from different liver areas were removed and immersed in phosphate Sørensen buffer solution (pH 7.4) containing 2.5% glutaraldehyde. For TEM, multiple 1-mm3 pieces of liver were routinely processed and embedded in epoxy resin. Ultrathin sections, stained with uranyl acetate, were examined under a Jeol JEM-1010 electron microscope. The sections were not stained with lead citrate as usual, because this could interfere with the gold nanoparticles and make their detection very difficult.

## Results

With regard to the *endovascular closed procedure* (Fig 1A), liver venous vasculature blockade was confirmed and no relevant electrocardiographic changes were observed. Blood pressure decreased during circulatory arrest (to 30 mmHg), but quickly recovered (in less than 1 min) when the balloons were deflated. The AST levels increased to 150 IU (higher than with the open procedure), while ALT also increased to 70 IU (3-fold higher than in the open procedure). On day 5, the transaminases levels tended to normalize, with concentrations of 125 and 57 IU, respectively.

The *surgically closed model* (Fig 1B) sealed the entire organ after vascular inflow and outflow perfusion occlusion. Both systolic and diastolic blood pressures decreased gradually (approximately 30–50%) when the liver vasculature was completely excluded. The pressure was partially augmented (14–27%) by plasmid injection. The lowest pressure level was reached 5 minutes (time lapse for gene entry to the cell) after injection from 87/61 ± 9/6 mmHg (basal) to 42/33 ± 6/4 mmHg. Once the liver was vascularized, blood pressure recovered in less than 5 minutes, showing no significant differences versus the basal values one minute after revascularization. No relevant changes were detected in the ECG (electrocardiogram) tracing. Oxygen saturation was maintained above 98% during the entire operation. The intervention produced an increase in AST levels (to 72.4 ± 29.9 IU) one hour after injection, and this was completely reverted 24 hours after injection (32.4 ± 11.5 IU). The plasma ALT levels remained unaltered in the evaluated samples.

## Protein expression in liver tissue

**Fig 2:**
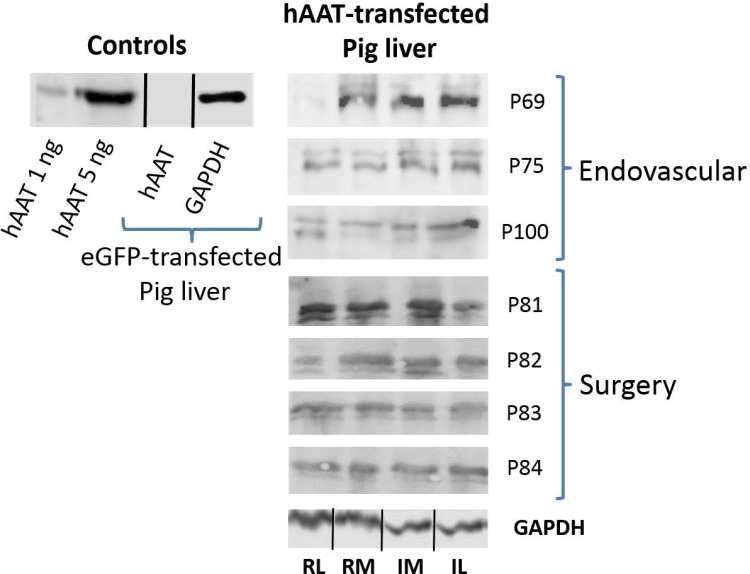
hAAT protein expression in liver tissue. hAAT protein expression in liver tissue is represented. The part at left corresponds to controls. Positive controls: pure hAAT protein (1 and 5 ng). Negative control: homogenate from eGFP transfected pig liver tissue (50 μg of total protein). The part at right in the figure represents hAAT protein presence in liver tissue after both the endovascular and surgery closed perfusion procedures. Translation in each liver lobe is represented. Equivalent amounts of total protein from distal and proximal tissue samples of each liver lobe were mixed. First letter: R = right, L = left; Second letter: M = medial, L = lateral; The id of pigs follows the internal nomenclature employed in our laboratory.

Semi-quantitative analysis by Western blot (Fig 2, original WBs shown at S1–S3 Figs) showed hAAT protein expression in pig liver to be approximately 10-fold lower than in human liver tissue.

**Fig 3:**
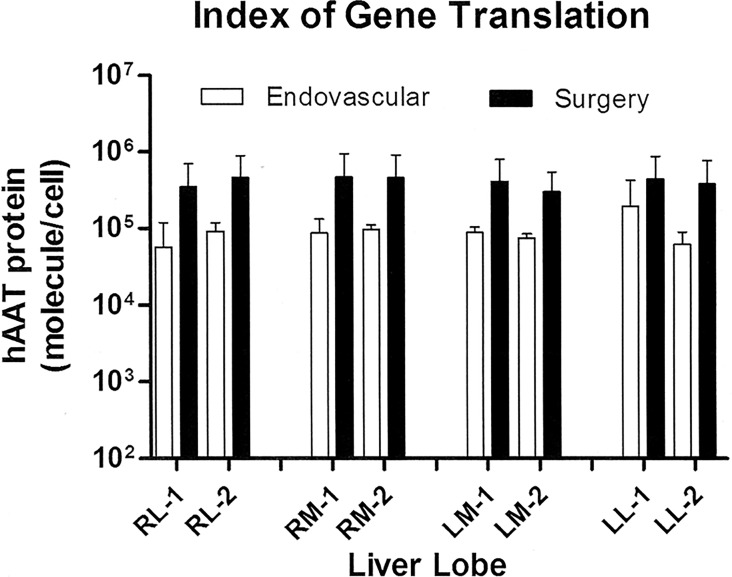
hAAT gene translation index in liver tissue. Fig 3 represents the gene translation index per cell, calculated per ELISA, after both the endovascular and surgery closed perfusion procedures. Translation in each liver lobe is represented (mean ± SD). First letter: R = right, L = left; Second letter: M = medial, L = lateral; Number: 1 = upper, 2 = lower. Statistical analysis: linear mixed model with pig as random factor, hAAT protein as response variable, and group, liver lobe and their interaction as explicative variables.

Quantitative expression of hAAT (Fig 3) was registered as the number of hAAT molecules per cell, considering the average amount of total protein in a mammalian cell [26–28]. The endovascular- and surgery-mediated closed inflow models yielded a mean protein translation index of 10^5 and 4x10^5 protein molecules per cell in tissue, respectively. The protein translation achieved with surgery was greater, though without reaching statistical significance (p = 0.209). As expected, since the whole organ was targeted, similar hAAT protein expression levels were detected in the different lobes of pig liver transfected with the two closed models, though increased expression (p = 0.033) was observed in right medial lobes.

## Protein expression in plasma

The presence of hAAT protein was also quantified in blood samples collected at different timepoints during the two weeks. The serum levels of hAAT protein (Table 1) were lower than expected, compared with the reference levels, and much lower than the concentration considered to be therapeutic (0.8 mg/ml). Although the observed dispersion was high, the endovascular-mediated closed procedure resulted in higher levels than the surgical model—no significant differences being found (p = 0.137).

**Table 1:** hAAT protein concentration in plasma on day 14.

hAAT protein concentration in plasma		
Sampling time (day)	Endovascular (ng/ml)	Surgery (ng/ml)
1	44.5 ± 19.2	13.5 ± 17.1
2	28.9 ± 2.9	16.3 ± 18.9
5	50.0 ± 46.9	9.4 ± 11.7
7	27.1 ± 38.4	18.1 ± 36.3
10	20.6 ± 28.5	31.5 ± 38.9
14	28.2 ± 30.1	5.8 ± 11.6

Table 1. hAAT protein expression in plasma: The table shows protein expression in plasma at different times after DNA perfusion by the endovascular and surgically closed procedure, indicated as hAAT concentration in ng/ml, calculated by ELISA (mean ± SD). Statistical analysis: Linear regression with the area under the curve (AUC) as response and group as explicative variable.

## Comparison with standards

**Fig 4:**
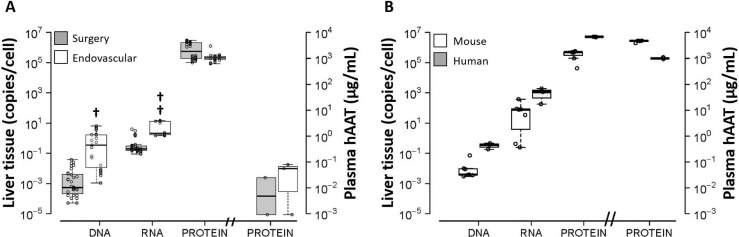
Pig hAAT gene decoding by catheterization and surgical closed procedure versus standards. The average copy contents (mean ± SD) of hAAT DNA, RNA and protein (tissue and plasma) in pigs after closed liver hydrofection (A) are compared with those obtained in standards (human liver and hydrodynamically transfected mouse liver, B). Tissue indexes are shown as copy number per cell in each case. Protein expression in plasma is expressed as concentration (μg/ml). Statistical analysis: Linear mixed model with expression level as response variable and sample type (DNA, RNA, protein or plasma), species and their interaction as explicative variables; statistically significant interaction between pig and protein in serum (p < 0.001). †: p<0.05 (p = 0.027); ††: p<0.01 (p = 0.007).

The whole hAAT gene decoding process in hydrofected pigs (Fig 4A) was compared with two standards: hAAT transfected mice and normal human liver (Fig 4B). hAAT DNA, RNA and protein were quantified in liver tissue samples, and data were represented as copies per normalized cell. Transfected mouse and human liver segments exhibited similar behavior in relation to the hAAT gene decoding process. Interestingly, the hAAT decoding process of the standards showed perfect linear correlation (r = 0.99) in the entire process. In both cases, therapeutic levels of protein in serum were observed, thus indicating optimum performance.

In contrast, and despite the marked presence of hAAT in liver tissue, the presence of protein in pig serum was very low in all cases, suggesting a defect in the protein secretion step. This effect was assessed and quantified using a regression model including an interaction between species and sample type. This interaction considers a different concentration of each molecular species and different decoding efficiency in each animal model, allowing us to test different decoding patterns among species. The interaction was statistically significant between pig and protein in serum (p<0.001), with estimated levels in pigs 4.24 units lower on the log-scale compared to human levels. This phenomenon occurred in the two closed models of hydrofected pigs. The amount of delivered DNA in endovascular-closed hydrofected pigs (1 gene copy/cell) was close to that recorded in humans, higher than in mice standards, and higher (p = 0.027) than the amounts obtained with the surgical procedure. The RNA transcription index was lower in pigs than in reference standards, the endovascular procedure mediating higher rates (p = 0.007) than surgery. As aforementioned, no difference regarding protein presence in tissue and plasma were found between both procedures. Raw molecular data of hAAT decoding process in pig, mouse and human are shown within S1 Table.

## Distribution of hAAT protein expression within liver tissue

**Fig 5:**
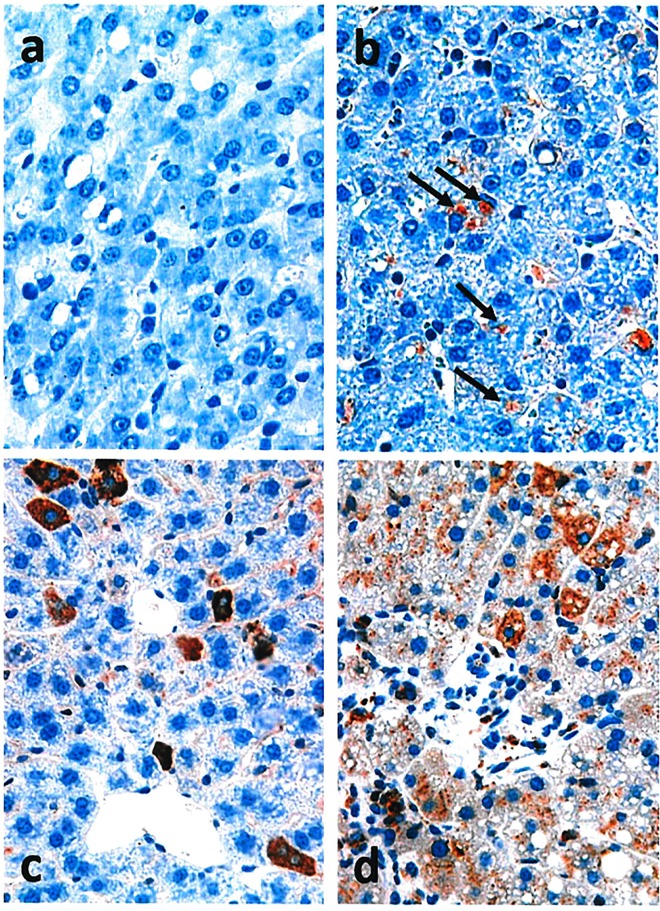
hAAT Immunohistochemistry. Non-transfected pig liver hAAT staining (a; 40x) versus transfection employing endovascular closed catheterization (b; 40x). hAAT protein staining in liver sections of transfected mice is shown (c; 40x); human liver hAAT immunostaining can be observed (d; 40x). Black arrows indicate representative specific immunohistochemical reaction against hAAT protein in pig samples. Tissue was counterstained with hematoxylin.

Hydrofected pig liver samples were compared with human livers and hAAT hydrodynamically transfected mice under optimal conditions (standards) to evaluate liver distribution of hAAT protein by immunohistochemical analysis. Tissue structure proved to be normal in all cases (Fig 5). A control sample of pig liver tissue transfected with eGFP gene is shown in Fig 5A. In pig liver samples hydrofected with hAAT gene (Fig 5B), hAAT protein was detected with an inhomogeneous distribution in all cases. The protein was expressed in less than 5% of the cells.

In standard liver sections from transfected mouse (Fig 5C), protein expression was higher but likewise present in a reduced number of cells (approximately 5–10%).

In both pig and mouse hydrofected livers, the hAAT stained cells were preferentially located in the central perivenous area. In contrast, the distribution of hAAT staining in humans (Fig 5D) was homogeneous throughout the tissue, with markedly greater expression in cells preferentially located in the periportal area.

## Discussion

Intravascular hydrodynamic procedures for transferring genes are safe and have therapeutic advantages in small animal models. In our study, gene hydrofection efficiency in pigs, mediated by closed inflow models, was compared regarding gene delivery and RNA and protein expressions calculated by molecular analyses versus two controls that reached therapeutic plasma levels of the protein (hydrodynamically transfected mice and *ex vivo* human liver tissue). Our results show that the retrograde hydrofection of a naked plasmid bearing the whole genomic hAAT gene is highly efficient in expressing the protein in tissue of entire pig liver with both the endovascular and surgery closed inflow procedure, as indicated by quantitative PCR, Western blot, ELISA and immunohistochemical analysis.

The correlation between high pressures and hydrofection efficiency has not been well established. In our hands, mild hydrodynamic conditions could offer some advantages. In this sense, closed procedures with lower hydrodynamic pressures resulted in better transfection outcomes. In our experience, the open procedure employed in previous studies [30] achieved an average pressure (measured in portal vein) of 95 mmHg during the injection of plasmid solution, and mediated a protein translation index in tissue of 10^4 copies/cell. In contrast, when employing the surgery closed procedure and retrovenous injection to transfect the whole liver, the average pressure achieved was lower than 30 mmHg but mediated a translation index in tissue of 4x10^5 copies/cell. The pressure data from surgical closed hydrofection are in agreement with those obtained by Fabre et al. when injecting the DNA solution through a suprahepatic vein with blocked venous outflow. The use of automatized systems of injection such as those described by Fabre et al. [17] and Kamimura et al. [19] could contribute to control and establish the optimal conditions of hydrodynamic gene transfer to secure maximum efficiency.

The expression of human proteins in pigs has not achieved levels close to those considered to be therapeutic in any case [15, 19]. In the present study, protein translation indexes in tissue achieved noticeable levels, but the presence of protein in plasma was low. In this sense, Kamimura et al. [19] demonstrated the presence of protein within liver tissue by immunohistochemistry. These authors did not quantitatively evaluate gene expression, but the intensity of reaction was greater than that observed by us. In this work, the best translation indexes were obtained by the closed surgical procedure, where the hepatic artery had been clamped (surgery 4x10^5 copies/cell > endovascular closed procedure 10^5 copies/cell > endovascular open procedure 2x10^4 copies/cell). The slight differences in tissue protein expression are not trivial: our goal is to reach the successful levels obtained with the hydrodynamic procedure in mice (10^6 molecules/cell). The catheterization open procedure yielded a protein translation index of 2x10^4 molecules/cell. The catheterization closed procedure obtained 10^5 molecules/cell (5-fold higher than in the open model). The surgery closed procedure mediated 4x10^5 molecules/cell (4-fold and 20-fold higher than in closed and open catheterization, respectively). This implies only 2.5-fold lower levels than the successful therapeutic levels achieved in mice (10^6 molecules/cell).

However, plasma presence of protein could not be detected, supporting the uncoupling between intracellular expression and plasmatic protein concentration. This phenomenon occurred in all pig experiments, with both the endovascular closed inflow and surgery procedures. The low protein levels obtained in plasma suggest that the low-end performance limiting hydrofection translation to the clinical setting must be studied in some step between the protein translation and secretion processes. The reason for such deficient protein secretion remains to be clarified. Considering the differences between species, impaired secretion could be due to inappropriate glycosylation of the protein or to differences between the exportation sequences of the human and pig hAAT proteins, as there is a difference of 5 amino acids in signal peptide between pig and human proteins [31].

However, the enormous potential clinical applications and low risk of adverse effects of endovascular hydrofection as now widely used in clinical practice for other purposes, and of surgery for liver isolation as currently used in liver transplantation, justify our detailed evaluation of the different steps of the information decoding system. This study allowed us to observe noticeable expression of foreign protein in liver tissue and could shed some light on the process responsible for the aforementioned plasma protein expression inefficiency in pigs, since it has been previously observed that gene transcription does occur [23].

**Fig 6:**
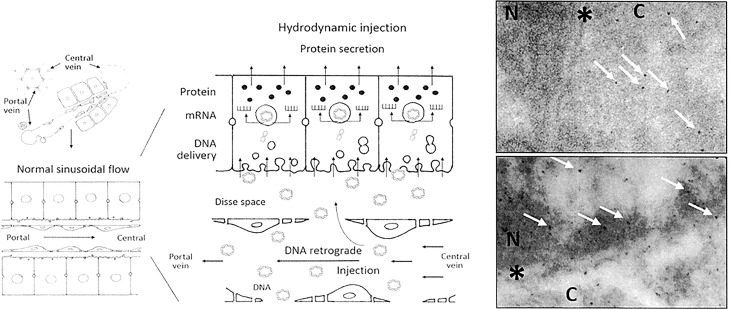
Histological and cellular scheme of hydrofection and nanoparticles distribution within liver tissue. The schematic area (left) provides a representation of how hydrodynamic retrograde injection, under the described conditions, mediates greater separation between endothelial cells and their fenestrations, as well as enlargement of the Disse space and the formation of massive endocytic vesicles. These changes result in DNA molecule entry into the liver cell nucleus, where DNA transcription to RNA occurs. Then, mRNA is released into the cell cytoplasm, where it is translated to protein. Finally, after the necessary modifications, the mature protein, if to be secreted, is released out of the tissue. The image at right provides a transmission electron microscopy view of the nuclear compartment of hepatocytes after transferring 4 nm and 15 nm gold nanoparticles by the surgery closed procedure (lower panel) and open procedure (upper panel). Nanoparticles only reach the nucleus with more energetic conditions of the open procedure. N: nucleus; C: cytoplasm; *: nuclear envelope. White arrows: nanoparticles 4–8 nm in diameter.

The radiological endovascular approach has several strengths as a therapeutic procedure. Firstly, the hydrofection procedure mediates good results regarding gene delivery and expression in the liver, not significantly different from those of the surgical procedure. Moreover, this acute pressure-mediated procedure is well tolerated by the animals. Secondly, successful gene delivery is obtained by the closed inflow procedure, yielding 1 copy/cell. Furthermore, and although the transcription index was lower than expected with respect to the reference models, the protein levels obtained in tissue were within the expected range of approximately 1x10^5 copies/cell. For accomplishing the decoding process, the transferred DNA must access the cell nucleus with availability to be transcribed. Fig 6 (panel at left) provides an illustrative scheme that suggests the process of gene delivery after hydrodynamic retrograde injection. Hydrodynamic retrograde transfer mediates greater separation between endothelial cells and their fenestrations, with enlargement of the Disse space and the formation of massive endocytic vesicles [32–34]. These changes result in DNA molecule entry into the liver cell nucleus. In order to elucidate the process of DNA delivery, we performed two exploratory experiments transferring gold nanoparticles of 4 nm and 15 nm, as previously described [23, 24, 35], observing their tissue distribution under the electron microscope. For these experiments, we employed the surgically closed procedure (Fig 6, lower right panel) that targets the whole sealed liver, and another more hydrodynamically energetic procedure [20] (Fig 6, upper right panel), in which the same injection conditions (200 ml; 20 ml/s) were applied in a single lobe. Whereas many 4 nm nanoparticles could freely reach the nucleus of the hepatocytes after being transferred under the latter energetic conditions, we failed to find them when the nanoparticles were transferred under the milder conditions of the surgically closed procedure. Particles reached the nuclear envelope but were unable to cross it. Although DNA entrance to cells is not well understood yet, our data suggest that DNA entrance appears to be mediated by a diameter-conditioned passive permeation process.

Although more invasive than the catheterization closed procedure, the surgically closed model allows control of complete sealing of the liver in order to evaluate the benefits derived. In addition, the surgical procedure has a specific gene therapy application in liver transplantation, since the surgical procedure employed is identical and could play an important role in the early onset of response modulation to prevent graft rejection, employing immunosuppressive genes such as IL10 [36].

In this setting, liver pig models allow us to evaluate the process of gene decoding within liver tissue, but it is not possible to solve the inter-species differences observed during protein exportation when a human gene is employed. The standards (human livers and hydrodynamically transfected mice) showed a linear relationship (r = 0.99) along the gene decoding process (DNA, RNA and protein) in tissue, but not the pig models. Unfortunately, the greater amount of gene delivered in the endovascular closed model did not result in proportionally higher hAAT RNA levels. This could be due to partial kidnapping of transferred gene within other organelles such as mitochondria, as previously reported [37]. The phenomenon could also be explained if excess gene delivery respect to the transcription factors occurs within the cell, mainly assuming that only a reduced number of cells (average of 10% immunostained cells) could have been successfully transfected. The apparent gene delivery observed (1 plasmid copy delivered per cell) could in fact represent a real value of 10 copies in each transfected cell, where only 10% of the total cells have actually been transfected. In any case, this effect was compensated in translation, since the protein levels were very similar to the standards in liver tissue. Our immunohistochemical results support the abovementioned concept, since the proportion of liver pig cells marked positively with anti-hAAT antibody was approximately 3–5%, thus supporting differential gene delivery in the cell population. Therefore, the main obstacle against efficient hydrofection in pigs must be located between translation and secretion, which could be species-related.

In summary, *endovascular and surgical closed models for hydrofection-mediated gene therapy procedure allow successful hAAT gene delivery and tissue protein expression in pigs*. The inefficiency of protein secretion in pig liver could be species-related and might be solved by employing e*x vivo* human liver segments, considering the use of a natural human gene. This study reinforces the potential clinical application of hydrofection in many fields and diseases, such as hAAT deficiency or a broad range of other metabolic disorders.

## Supporting Information

S1 Fig Original Western blot showing the hAAT protein expression in control liver tissue. hAAT protein expression in control liver tissue is represented. Positive controls: pure hAAT protein (1 and 5 ng). Negative control: homogenate from eGFP transfected pig liver tissue (50 μg of total protein). Ø: empty.
(TIF)

S2 Fig Original Western blot showing the hAAT protein expression in liver tissue transfected with hAAT by endovascular procedure. hAAT protein expression in liver tissue transfected by endovascular procedure is represented. Translation in each liver lobe is represented. Equivalent amounts of total protein from distal and proximal tissue samples of each liver lobe were mixed. First letter: R = right, L = left; Second letter: M = medial, L = lateral. The id of pigs follows the internal nomenclature employed in our laboratory.
(TIF)

S3 Fig Original Western blot showing the hAAT protein expression in liver tissue transfected with hAAT by surgical procedure. hAAT protein expression in liver tissue transfected by surgical procedure is represented. Translation in each liver lobe is represented. Equivalent amounts of total protein from distal and proximal tissue samples of each liver lobe were mixed. First letter: R = right, L = left; Second letter: M = medial, L = lateral. The id of pigs follows the internal nomenclature employed in our laboratory.
(TIF)

S1 Table Raw unprocessed data of hAAT gene decoding process. Individual data of each molecular specie from each individual sample is shown. DNA, RNA and protein copies per cell in liver tissue and hAAT protein concentration in plasma (μg/ml) at different sampling times are presented.
(XLSX)
