# Supplementary figures and images for: Studying Closed Hydrodynamic Models of “In Vivo” DNA Perfusion in Pig Liver for Gene Therapy Translation to Humans
Source: PLoS One. 2016 Oct 3;11(10):e0163898. doi: 10.1371/journal.pone.0163898 (PMC5047531; doi:10.1371/journal.pone.0163898)

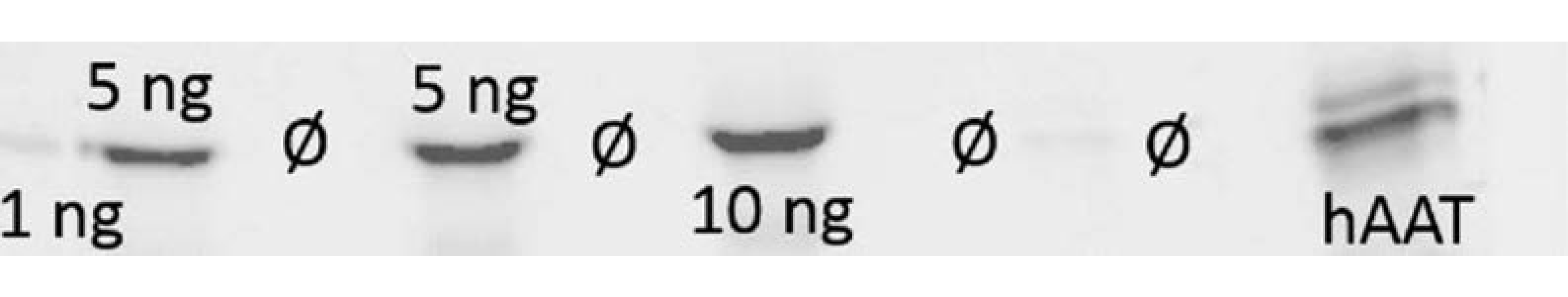

Supplement: S1 Fig — hAAT protein expression in control liver tissue is represented. Positive controls: pure hAAT protein (1 and 5 ng). Negative control: homogenate from eGFP transfected pig liver tissue (50 μg of total protein). Ø: empty. (TIF) [file pone.0163898.s001.tif]

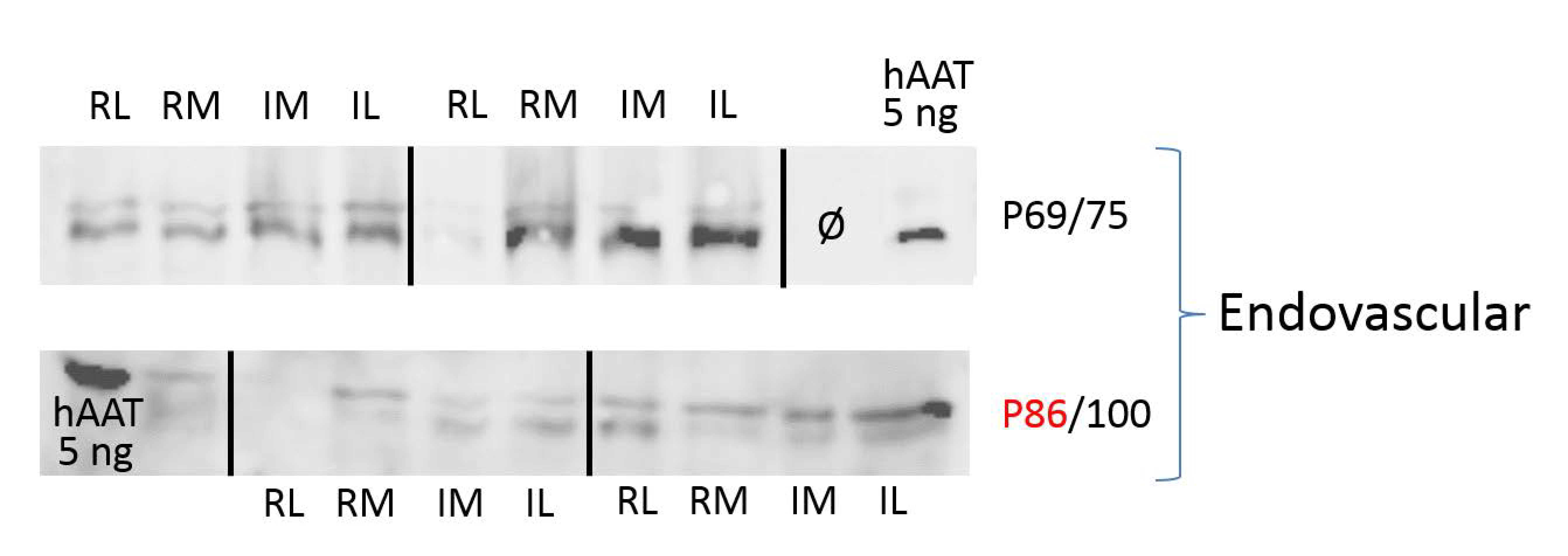

Supplement: S2 Fig — hAAT protein expression in liver tissue transfected by endovascular procedure is represented. Translation in each liver lobe is represented. Equivalent amounts of total protein from distal and proximal tissue samples of each liver lobe were mixed. First letter: R = right, L = left; Second letter: M = medial, L = lateral. The id of pigs follows the internal nomenclature employed in our laboratory. (TIF) [file pone.0163898.s002.tif]

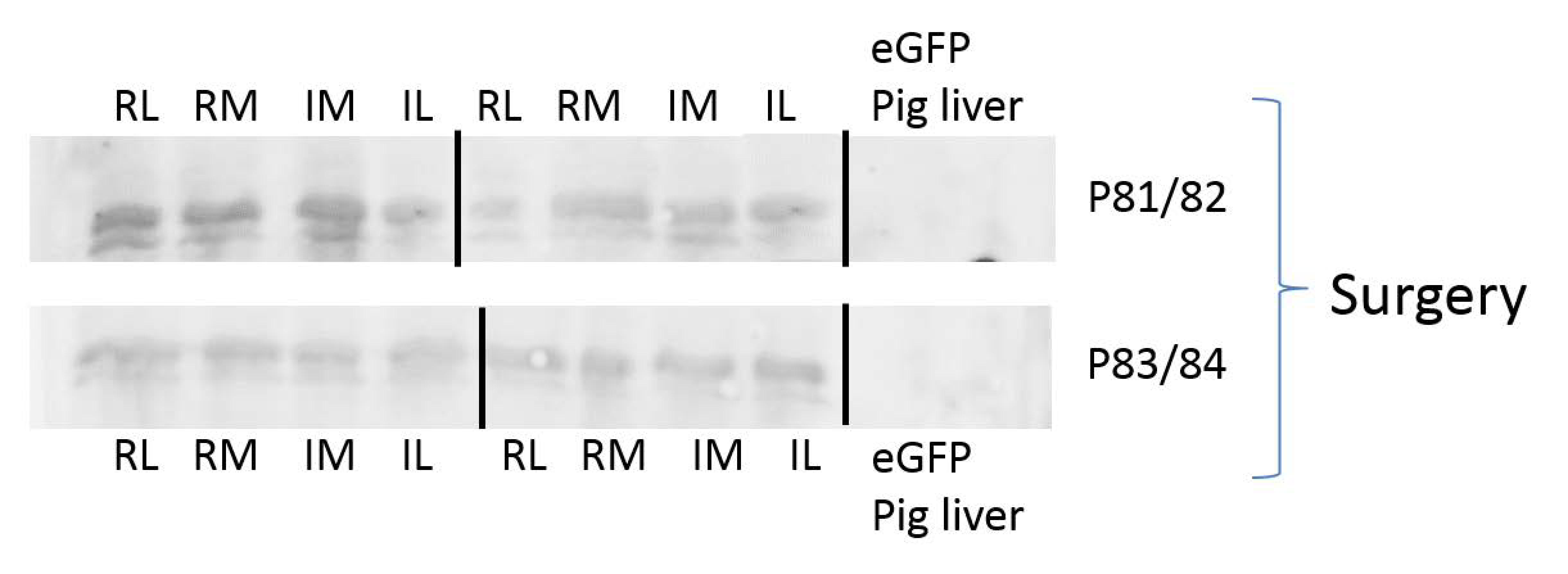

Supplement: S3 Fig — hAAT protein expression in liver tissue transfected by surgical procedure is represented. Translation in each liver lobe is represented. Equivalent amounts of total protein from distal and proximal tissue samples of each liver lobe were mixed. First letter: R = right, L = left; Second letter: M = medial, L = lateral. The id of pigs follows the internal nomenclature employed in our laboratory. (TIF) [file pone.0163898.s003.tif]
